# Protective effects of a multicomponent toxin binder and organic acid blend on feed efficiency, oxidative status, hepatic histology, and jejunal immune–antioxidant responses in broilers co-challenged with aflatoxin B_1_ and *Clostridium perfringens*

**DOI:** 10.1016/j.psj.2026.106546

**Published:** 2026-01-29

**Authors:** Maryam Karimi Zandi, Hassan Shirzadi, Hossein Ali Ghasemi, Mohammad Amir Karimi Torshizi, Kamran Taherpour, Enayat Rahmatnejad

**Affiliations:** a Department of Animal Science, Faculty of Agriculture, Ilam University, Ilam, Iran; b Department of Animal Science, Faculty of Agriculture and Environment, Arak University, Arak 38156-8-8349, Iran; c Department of Poultry Science, Faculty of Agriculture, Tarbiat Modares University, PO Box 14115-336, Tehran, Iran.; d Department of Animal Science, Faculty of Agriculture and Natural Resources, Persian Gulf University, Bushehr 75169, Iran

**Keywords:** Oxidative stress, Acidifiers, Toxin binder complex, Afaltoxin-fed broilers, Enteric pathogen

## Abstract

This study evaluated the effects of a multicomponent toxin binder (**MTB**) and an organic acid blend (**OAB**) on performance, immunity, oxidative status, liver histology, and jejunal inflammatory/antioxidant gene expression in broilers challenged with aflatoxin B_1_ (**AFB_1_**) and *Clostridium perfringens*. A total of 420 Ross 308 broilers were assigned to seven groups (6 replicates × 10 birds): Control (unchallenged), A (AFB_1_), AM (AFB_1_ + MTB), AMO (AFB_1_ + MTB + OAB), AC (AFB_1_+ *C. perfringens*), ACM (AFB_1_ + *C. perfringens* + MTB), and ACMO (AFB_1_ + *C. perfringens* + MTB+OAB). AFB_1_ (500 ppb) was provided throughout days 0–42; *C. perfringens* (1 × 10⁸ CFU/mL) was administered on days 15–24. AFB_1_ alone, and more markedly the AFB_1_+*C. perfringens* co-challenge, reduced body-weight gain and feed efficiency, increased hepatic superoxide dismutase activity and malondialdehyde level, enlarged central-vein diameter, upregulated jejunal NF-κB1, TNF-α, and IL-6, and downregulated hepatic total antioxidant capacity and jejunal NRF2 and SOD1 mRNA expression (*P* < 0.05). The co-challenge also lowered Newcastle disease antibody titers, reduced phytohemagglutinin-induced toe-web swelling, and increased the heterophil:lymphocyte ratio (*P* < 0.05). Although MTB attenuated several AFB_1_-related impairments, MTB+OAB provided superior protection under co-challenge, increasing hepatic total antioxidant capacity, lowering malondialdehyde, improving liver histoarchitecture (central-vein diameter), and normalizing the expression of immune and antioxidant genes toward control levels, alongside improvements in performance indices (*P* < 0.05). In conclusion, although co-exposure to AFB_1_ and *C. perfringens* caused greater detriments than AFB_1_ alone, adding OAB to MTB improved performance, oxidative, histological, and immunological outcomes, supporting MTB+OAB as a practical strategy for broilers under concurrent mycotoxin–enteric challenge.

## Introduction

Aflatoxin B_1_ (**AFB_1_**) is the most common and biologically active aflatoxin, causing hepatic injury, carcinogenesis, mutagenesis, and immunosuppression in animals (Mehrim and Salem, 2013; Cheng et al., 2023a). In the intestine, aflatoxins induce dysbiosis, trigger immune responses, and generate reactive oxygen species, leading to microstructural damage, reduced nutrient digestibility, and impaired growth (Gao et al., 2020; Mohammadi et al., 2025). A meta-analysis showed that each μg AFB_1_ per kg body weight per day reduces average daily gain by 0.13% in poultry and 0.74% in pigs (Choi et al., 2025). AFB_1_ also suppresses immunity, disrupting cellular immune function, increasing mitogen skin reactivity, and lowering antibody titers against Newcastle disease virus, influenza virus, and SRBC antigens (Lai et al., 2022). Hepatic conversion of AFB_1_ to AFB_1_-exo-8,9-epoxide further perturbs signaling pathways (Kozieł et al., 2021). Mechanistically, dysregulation of NF-κB (inflammatory) and NRF2 (antioxidant) pathways can promote oxidative stress, lipid peroxidation (e.g., malondialdehyde formation), and tissue injury (Cui et al., 2012; Cheng et al., 2023b). Consistently, AFB_1_ feeding reduced hepatic NRF2 expression and disrupted the antioxidant enzyme program (e.g., SOD, GPx) in broilers (Chen et al., 2024).

AFB_1_ may predispose to secondary infections by facilitating *Clostridium perfringens* colonization (Choi et al., 2025). *C. perfringens*, a normal inhabitant of the avian gut, commonly proliferates between 2–6 weeks of age under immune stress, causing necrotic enteritis with case fatality approaching 30% if untreated (Alkhulaifi et al., 2022; Gautam et al., 2024). Evidence indicates bidirectional exacerbation: aflatoxin worsens *C. perfringens*–induced enteritis, and *C. perfringens* aggravates aflatoxicosis (Abd El-Hamid et al., 2017; Cravens et al., 2013). These interactions underscore the need for strategies that attenuate both aflatoxicosis and necrotic enteritis.

Toxin binders are nutritionally inert feed additives that sequester mycotoxins in the gastrointestinal tract, limiting absorption (Kihal et al., 2022). In broilers under AFB_1_ challenge, binders can mitigate toxicosis and support health and immune function (Rashidi et al., 2020). New-generation multicomponent binders pair classical adsorbents with probiotic, toxin-degrading microorganisms. Spore-forming *Bacillus* spp. and *Bifidobacterium* spp. contribute enzymatic biotransformation of mycotoxins, competitively exclude pathogens, and secrete digestive enzymes, thereby stabilizing the microbiota and supporting epithelial integrity under challenge (Asadi et al., 2018; Nguyen et al., 2024). This combined adsorption–biotransformation design broadens detoxification and may help sustain performance during co-challenge.

Organic acids (acidifiers) improve gut health by lowering luminal pH, enhancing proteolytic activity and nutrient digestibility, modulating microbiota, stimulating beneficial bacteria, and suppressing pathogens (Dittoe et al., 2018; Papatsiros et al., 2014). Formic, propionic, and acetic acids decrease digesta pH and, in their undissociated form, disrupt pathogen metabolism (Abd El-Hack et al., 2024; Martínez et al., 2021), while citric acid can enhance Ca and P availability, likely via calcium chelation and phytate destabilization (Tugnoli et al., 2020). Given their antimicrobial action and potential to reduce aflatoxin activity, acidifiers are logical complements to toxin binders.

We hypothesized that, in broilers simultaneously exposed to AFB_1_ and *C. perfringens*, a multicomponent mycotoxin binder containing probiotic, toxin-degrading microorganisms would mitigate AFB_1_-associated disturbances, and that co-supplementation with an organic acid blend would further reduce pathogen burden and barrier injury, outperforming the binder alone. Because integrated evaluations under concurrent AFB_1_ and *C. perfringens* challenge remain scarce, particularly for oxidative stress and immune endpoints, this study assessed the effects of AFB_1_ alone and combined with *C. perfringens* on growth, immunity, serum and hepatic antioxidant status, liver histology, and jejunal inflammatory/antioxidant gene expression, and tested the efficacy of a multicomponent toxin binder (**MTB**) and an organic acid blend (**OAB**) to mitigate aflatoxicosis and necrotic enteritis.

## Materials and methods

### Birds and experimental treatments

The Animal Ethics Committee of Ilam University has approved this study (Approved number: IR.ILAM.REC.1404.019) and animal trials was conducted in compliance with the regulations stated in the ARRIVE guidelines 2.0 for the Care and Use of Experimental Animals. This study was conducted using 420 mixed-breed Ross 308 broiler chickens to evaluate the effects of MTB and OAB on growth performance, immune response, serum and liver tissue antioxidant status, liver tissue morphology, and the expression of inflammatory and antioxidant genes in jejunal tissue in broiler chickens co-challenged with AFB_1_ and *C. perfringens*. The experiment followed a completely randomized design with seven treatments and six replicates (10 birds per replicate). The sample size was determined based on standard statistical power calculations to ensure sufficient power (80%) to detect a meaningful difference between treatments at a significance level of 0.05. The birds were housed in four-tier battery cages and randomly assigned to experimental units before the start of the study. The experimental treatments were as follows: **Control** (basal diet without additives and without AFB_1_ and *C. perfringens* challenge); **A** (basal diet without additives, challenged with AFB_1_); **AM** (basal diet with MTB, challenged with AFB_1_); **AMO** (basal diet with MTB and OAB, challenged with AFB_1_); **AC** (basal diet without additives, co-challenged with AFB_1_ and *C. perfringens*); **ACM** (basal diet with MTB, co-challenged with AFB_1_ and *C. perfringens*); and **ACMO** (basal diet with MTB and OAB, co-challenged with AFB_1_ and *C. perfringens*). From the beginning of the experiment until its completion, the chickens were exposed to AFB_1_ at a concentration of 500 ppm. Starting on day 15, the birds were also challenged with *C. perfringens* (ATCC No. 13124; Pasteur Institute of Iran, Tehran, Iran), with each bird orally inoculated daily with 1 mL of a *C. perfringens* suspension containing 1 × 10⁸ CFU/mL for 10 consecutive days (days 15–24). MTB and OAB were added to the basal diet at a concentration of 0.2% from the beginning of the experiment through its conclusion.

The MTB (Magnotox®) used in this study was a multicomponent product containing aluminosilicates, activated charcoal, yeast cell walls, and fungal toxin-degrading microorganisms (*Bacillus subtilis, Bacillus licheniformis, Bacillus coagulans*, and *Bifidobacterium bifidum*). The OAB (Vivacid®) included formic, acetic, propionic, and citric acids. Both additives were sourced from a commercial supplier (Vivan Company, Mashhad, Iran). The basal diets for the starter (0–10 days), grower (11–24 days), and finisher (25–42 days) periods were formulated according to the Ross 308 Broiler Breeding Guide. The ingredient composition and chemical analysis of the basal diets are detailed in Table 1.

**Table 1:** Feed ingredients and nutrient composition of basal diets (as fed basis).

Item (% of the diet)	Starter	Grower	Finisher
	(0-10 d)	(11-24 d)	(25-42 d)
Corn grain	53.75	58.45	63.87
Corn gluten meal	5.24	5.81	4.53
Soybean meal (44% CP)	32.43	28.00	24.21
Corn oil	2.10	2.14	2.15
Vitamin-mineral premix^1^	0.50	0.50	0.50
Dicalcium phosphate	2.12	1.69	1.39
Calcium carbonate	0.89	0.66	0.61
NaCl	0.16	0.19	0.18
NaHCO_3_	0.37	0.34	0.34
L-Lysine-HCL	0.48	0.42	0.41
DL-Methionine	0.39	0.33	0.32
L-Threonine	0.19	0.15	0.14
L-Arginine	0.17	0.15	0.16
L-Valine	0.09	0.05	0.07
Rice^i^	0.72	0.72	0.72
Sand^2^	0.40	0.40	0.40
Nutrient composition (% of diet, unless otherwise stated)			
Metabolizable energy (Kcal/kg)	2,975	3,050	3,100
Crude protein	23.0	21.5	19.5
Linoleic acid	1.31	1.39	1.50
Crude fiber	3.59	3.39	2.23
Calcium	0.95	0.75	0.65
Available phosphorus	0.50	0.42	0.36
Sodium	0.18	0.21	0.18
Potassium	0.83	0.76	0.69
Chlorine	0.23	0.23	0.23
DCAB^3^ (mEq/kg)	226	207	191
SID^4^ lysine	1.32	1.18	1.08
SID arginine	1.40	1.27	1.17
SID threonine	0.88	0.79	0.72
SID methionine	0.71	0.64	0.60
SID methionine + cysteine	1.00	0.92	0.86
SID tryptophan	0.21	0.19	0.17
SID leucine	1.87	1.82	1.65
SID isoleucine	0.83	0.77	0.69
SID valine	1.00	0.91	0.84

1 Supplied per kilogram diet: vitamin A, 9000 IU; vitamin D3, 2000 IU; vitamin E, 18 IU; vitamin K3, 2 mg; riboflavin, 6.6 mg; pantothenic acid, 10 mg; pyridoxine, 3 mg; folic acid, 1 mg; thiamin, 1.8 mg; B12, 15 µg; biotin, 0.1 mg; niacin, 30 mg; choline, 500 mg; Se, 0.2 mg; I, 1 mg; Cu, 10 mg; Fe, 50 mg; Zn, 85 mg; Mn, 100 mg.

2 The basal diets were supplemented with a multi-component toxin binder (Magnotox®) and/or an organic acid blend (Vivacid®) at 0.2% through substitution of sand to formulate the respective experimental diets.

3 Dietary cation anion balance.

4 Standardized ileal digestible

i To prepare the diets of the aflatoxin B1 (AFB_1_) treatments, rice was completely replaced with aflatoxin-contaminated rice (69.811 mg/kg), previously cultured with *Aspergillus parasiticus* [PTCC No. 5286; Iranian Research Organization for Science and Technology (IROST), Tehran, Iran].

### Growth performance and sampling

Body weight and feed intake per cage were recorded at the end of each phase (days 10, 24, and 42). From these data, average daily gain (**ADG**), average daily feed intake (**ADFI**), and feed efficiency (**FE** = ADG/ADFI) were calculated for each phase and for the overall period. Performance outcomes were corrected for mortality within each treatment.

### Humoral and cellular immune response

At 28 days of age, two birds per replicate cage with body weights approximating the cage mean were selected, and 5 mL of blood was collected from the wing vein. Immediately after venipuncture, blood smears were prepared and stained with Wright–Giemsa. Heterophils and lymphocytes were counted under a light microscope to calculate the heterophil-to-lymphocyte ratio (**H/L**). For serum separation, the remaining blood was allowed to clot at room temperature, followed by centrifugation at 2,000 × g for 15 minutes at 4°C. The serum was aliquoted into sterile tubes and stored at −20°C until humoral immunity assays (Zhao et al., 2024). Antibody titers against Newcastle disease virus (**NDV**) and avian influenza virus (**AIV**) were measured as described by Sedaghat et al. (2025). Basophilic cutaneous hypersensitivity, used as an indicator of cellular immunity, was assessed by measuring the response to 2,4-dinitro-1-chlorobenzene (24 hours after application to a featherless area) and phytohemagglutinin-P (24 and 48 hours after application between the toe webs), following methods described previously (Sedaghat et al., 2025; Sedaghat and Torshizi, 2017).

### Antioxidant status of serum and liver tissue

The remaining serum samples collected at 28 days of age were analyzed for total antioxidant capacity (**TAC**) and malondialdehyde (**MDA**). To assess hepatic antioxidant status, the right half of the liver was excised, rinsed in PBS, placed in PBS, and stored at −20°C until analysis (Zhao et al., 2024). A 10% (w/v) liver homogenate was prepared; the supernatant was obtained by centrifugation at 1,000 × g for 10 min at 4°C. Protein (cat. A045-2-2), TAC (A015-1-2), superoxide dismutase (**SOD**; A001-2-2), glutathione peroxidase (**GPx**; A005-1-2), and MDA (A003-1-2) were quantified using commercial kits according to the manufacturer’s instructions (Nanjing Jiancheng Bioengineering, Nanjing, China).

### Liver tissue morphology

The remaining liver tissue (day 28) was fixed in 10% formalin solution for 48 hours after washing with PBS. The tissue was then processed according to the method outlined by Beyari et al. (2024). Briefly, to remove formalin, the samples were immersed in distilled water and dehydrated with ethyl alcohol. After immersion in xylene, the samples were embedded in paraffin. Approximately 6 μm thick sections were prepared using a rotary microtome (Leica Microtome Model: Jung RM2045). A container with PBS solution at 45°C was placed under the microtome cutting area to prevent sample wrinkling. A clean slide immersed in water was placed under the sample to ensure adhesion. The samples were stained with hematoxylin and eosin, and hepatocyte diameter, hepatocyte nuclear diameter, and central vein diameter (**CVD**) were measured using an optical microscope (Olympus BX53, Tokyo, Japan) connected to a microscope digital camera (Olympus DP72, Olympus NV, Aartselaar, Belgium) and ImageJ software (version 1.52 v, Rawak Software Inc., Stuttgart, German). At 42 days of age, two birds per experimental unit (approximately the cage mean weight) were processed as above, and the same morphometric parameters were assessed.

### Expression of immune and antioxidant genes in jejunal tissue

On day 28, two birds per replicate (approximating the cage mean body weight) were slaughtered, as described previously. After isolating the jejunum, a mid-jejunum segment was excised, luminal contents were removed, and the tissue was rinsed with PBS. A small portion of the mid-segment was placed in RNAlater and transported to the laboratory. From each cage, equal masses (50 mg) from each of the two jejunal samples were pooled, and total RNA was extracted using TRIzol reagent (Invitrogen, Carlsbad, CA, USA) according to the manufacturer’s instructions. RNA purity and concentration were assessed with a NanoDrop 2000 spectrophotometer (Thermo Fisher Scientific, Waltham, MA, USA). cDNA was synthesized from RNA using a reverse transcription kit (Servicebio, Wuhan, China).

Primers for the target genes [nuclear factor kappa B subunit 1 (**NF-κB1**); tumor necrosis factor alpha (**TNFɑ**); interleukin 6 (**IL6**); interleukin 10 (**IL10**); nuclear factor erythroid 2 like 2 (**NRF2** or **NFE2L2**); superoxide dismutase 1 (**SOD1**); glutathione peroxidase 1 (**GPX1**)] were designed in Allele ID v7.5 and validated for specificity using NCBI Primer-BLAST (Table 2). Quantitative real-time PCR was performed on a StepOnePlus system (Applied Biosystems) using SYBR Green Master Mix in 25-µL reactions run in triplicate, as previously described (Shirzadi et al., 2024). Cycling conditions were: 95°C for 5 min, followed by 40 cycles of 95°C for 15 s and 60°C for 45 s. Relative mRNA abundance was calculated by the 2^−ΔΔCt^ method, with β-actin (ACTB) as the reference gene.

**Table 2:** Gene special primers used in the real-time quantitative reverse transcription PCR.

Gene^1^	Accession No.	Primer sequence (5^0^−3^0^)^2^	Length (nt)
Housekeeping gene			
ACTB	NM_205518.2	F: GAATCCGGACCCTCCATTGT	158
		R: AATCCTGAGTCAAGCGCCAA	
Immunity genes			
NF-κB1	XM_046939918.1	F: ACTTCCTGGTGCTTCTAGTGAA	179
		R: CTGCGTTGACTGTGCATACTCC	
TNFɑ	XM_040694846.2	F: ACCGCCACCGTCACCGCTCT	146
		R: GGTTTGTCCCTCCGCGCCACCT	
IL6	NM_204628.2	F: TTCAGCAATGGCAACAGCAATG	156
		R: ATAGCAACAAGCGTCGTATTTCAAC	
IL10	NM_001004414.4	F: GCTCTCACACCGCCTTGC	216
		R: ACTGCTTAACTGCTATCACTAACTCTC	
Antioxidant activity genes			
NRF2	NM_205117.1	F: ACATAGAGCAAGTTTGGGAAGAG	154
		R: GGTAACGAGTTGTAGTAATCATAGC	
SOD1	NM_205064.2	F: TTGTGGTGTAATTGGAATAGCC	159
		R: CAAGAACGCAGAGTAGTAATGAG	
GPX1	NM_001277853.3	F: AGTACATCATCTGGTCGCCG	118
		R: TTGATGGTCTCGAAGTGGCG	

1 **ACTB**, Beta Actin; **NF-κB1**, nuclear factor kappa B subunit 1; **TNFɑ**, tumor necrosis factor alpha; **IL6**, interleukin 6; **IL10**, interleukin 10; **NRF2** or **NFE2L2**, nuclear factor erythroid 2 like 2; **SOD1**, superoxide dismutase 1; **GPX1**, glutathione peroxidase 1.

2 F = forward primer; R = reverse primer.

### Statistical analysis

Data were analyzed in SAS (version 9.1; SAS Institute Inc., Cary, NC, USA) using the GLM procedure under a completely randomized design. Treatment means were compared with Tukey’s test at α = 0.05. Because the experimental challenges induced differences in initial body weight among groups, initial BW at the start of the grower and finisher phases was included as a covariate for performance analyses. The experimental unit for performance parameters was the cage, while for other individual-level parameters, each bird was considered the experimental unit. Data normality was assessed using the Shapiro-Wilk test prior to analysis.

## Results

### Growth performance

Table 3 presents performance outcomes during the starter and grower periods (to day 24). During the starter period, birds were challenged only with AFB₁. FE did not differ among treatments (P > 0.05). However, the AFB₁-challenged groups (A, AC) showed lower ADG than CON (P < 0.05). Supplementation with the MTB attenuated this effect, as AM and ACM achieved ADG comparable to CON. By contrast, combining MTB with OAB was not beneficial at this stage: AMO and ACMO exhibited lower ADG than CON (P < 0.05) and tended toward reduced ADFI (P = 0.09). During the grower period (days 15–24), birds were co-challenged with *C. perfringens* in addition to AFB₁. ADFI and FE remained unaffected (P > 0.05), but the dual challenge depressed ADG (P < 0.05). Both MTB alone and MTB + OAB improved ADG relative to the co-challenged, unsupplemented group (AC).

**Table 3:** Effects of dietary treatments on growth performance^1^ of broiler chickens challenged with aflatoxin B_1_ and *Clostridium perfringens* during the starter and grower periods.

Item^2^	Starter (0-10 d)			Grower (11-24 d)		
	ADG	ADFI	FE	ADG	ADFI	FE
Control	25.7^a^	30.2	0.853	60.3^a^	87.5	0.689
A	22.8^b^	29.1	0.788	56.7^ab^	84.3	0.672
AM	25.3^a^	29.9	0.850	59.1^a^	86.5	0.683
AMO	22.2^b^	27.5	0.809	60.0^a^	87.5	0.687
AC	23.0^b^	29.1	0.790	55.0^b^	84.6	0.650
ACM	25.7^a^	30.3	0.849	56.5^ab^	85.3	0.662
ACMO	22.5^b^	27.6	0.817	58.3^ab^	86.3	0.677
SEM	0.6	0.8	0.229	1.2	1.8	0.012
*P- value*	>0.01	0.09	0.19	0.03	0.79	0.12

a-c Means within a column without common superscript are significantly different at the level *P* < 0.05.

1 Abbreviations: **ADG**, average daily gain; **ADFI,** average daily feed intake, **FE**, feed efficiency.

2 Experimental groups**: Control,** unchallenged; **A**, aflatoxin B_1_ challenge; **AM**, aflatoxin B_1_ challenge + multi-component toxin binder (MTB); **AMO**, aflatoxin B_1_ challenge + MTB + organic acid blend (OAB); **AC**, aflatoxin B_1_ challenge + *Clostridium perfringens* challenge; **ACM**, aflatoxin B_1_ challenge + *Clostridium perfringens* challenge + MTB; **ACMO**, aflatoxin B_1_ challenge + *Clostridium perfringens* challenge + MTB + OAB; **SEM**, standard error of the mean.

Table 4 summarizes performance during the finisher period and the overall trial (0–42 d). In the finisher period, ADFI did not differ among treatments (P > 0.05). The AFB₁ + *C. perfringens* co-challenge impaired both ADG and FE (P < 0.05). MTB alone alleviated the reduction in ADG, whereas MTB + OAB was required to restore FE to values comparable to CON. Across the entire trial, ADFI was unaffected (P > 0.05). AFB₁ alone reduced ADG (P < 0.05); MTB mitigated this decline, and adding OAB did not further enhance ADG. Under co-challenge, both ADG and FE declined (P < 0.05). MTB by itself had limited impact on fully restoring these outcomes, whereas MTB + OAB was more effective, yielding FE comparable to CON.

**Table 4:** Effects of dietary treatments on growth performance^1^ of broiler chickens challenged with aflatoxin B_1_ and *Clostridium perfringens* during the finisher and entire rearing periods.

Item^2^	Finisher (25-42 d)			Overall (0-42 d)		
	ADG	ADFI	FE	ADG	ADFI	FE
Control	86.2^a^	158	0.546^a^	63.1^a^	104	0.607^a^
A	82.3^abc^	159	0.518^ab^	59.6^bcd^	103	0.577^ab^
AM	84.3^ab^	157	0.539^ab^	61.9^ab^	103	0.600^a^
AMO	85.0^a^	155	0.549^a^	61.7^abc^	102	0.605^a^
AC	76.0^c^	153	0.497^b^	56.3^d^	101	0.560^b^
ACM	77.9^bc^	156	0.499^b^	58.3^cd^	103	0.569^b^
ACMO	81.0^abc^	157	0.513^ab^	59.3^bcd^	103	0.578^ab^
SEM	2.1	4	0.013	1.1	2	0.009
*P- value*	0.01	0.98	0.03	<0.01	0.95	<0.01

a-d Means within a column without common superscript are significantly different at the level *P* < 0.05.

1 Abbreviations: **ADG**, average daily gain; **ADFI,** average daily feed intake, **FE**, feed efficiency.

2 Experimental groups**: Control,** unchallenged; **A**, aflatoxin B_1_ challenge; **AM**, aflatoxin B_1_ challenge + multi-component toxin binder (MTB); **AMO**, aflatoxin B_1_ challenge + MTB + organic acid blend (OAB); **AC**, aflatoxin B_1_ challenge + *Clostridium perfringens* challenge; **ACM**, aflatoxin B_1_ challenge + *Clostridium perfringens* challenge + MTB; **ACMO**, aflatoxin B_1_ challenge + *Clostridium perfringens* challenge + MTB + OAB; **SEM**, standard error of the mean.

### Cellular and humoral immune response

Table 5 presents cellular and humoral immunity outcomes. Antibody titer against AIV, skin thickness in response to DNCB, and toe-web thickness 24 h after PHA application were not affected by treatment (P > 0.05). In contrast, AFB₁ challenge decreased the lymphocyte percentage and increased both the heterophil percentage and the H/L ratio (P < 0.05). Supplementation with MTB alone and in combination with OAB improved these indices, with the combination showing the greater effect. Under AFB₁ + *C. perfringens* co-challenge, the antibody titer against NDV, toe-web response at 48 h post-PHA, and lymphocyte percentage declined, whereas heterophil percentage and H/L ratio increased (P < 0.05). MTB attenuated these adverse changes; adding OAB did not confer additional benefit for these immune parameters.

**Table 5:** Effects of dietary treatments on humoral and cell-mediated immune responses^1^ of broiler chickens challenged with aflatoxin B_1_ and *Clostridium perfringens*.

Item^2^	Antibody titers (Log_2_)		DNCB	PHA-P (mm)		Heterophil	Lymphocyte	H:L
	NDV	AIV	(mm)	24h	48h	(%)	(%)	
Control	5.83^a^	2.83	0.235	0.543	0.863^a^	43.0^bc^	56.0^a^	0.771 ^c^
A	5.00^ab^	2.67	0.195	0.506	0.814^ab^	54.5^ab^	41.0^b^	1.350 ^b^
AM	5.33^ab^	2.83	0.221	0.554	0.836^ab^	44.5^bc^	49.0^ab^	0.908^bc^
AMO	5.17^ab^	3.00	0.223	0.589	0.818^ab^	42.0^bc^	58.0^a^	0.727^c^
AC	4.83^b^	2.17	0.192	0.473	0.779^b^	64.5^a^	35.2^b^	1.949^a^
ACM	5.00^ab^	2.67	0.216	0.432	0.825^ab^	35.2^c^	58.0^a^	0.609^c^
ACMO	5.17^ab^	2.83	0.213	0.462	0.817^ab^	39.5^c^	58.0^a^	0.682^c^
SEM	0.20	0.28	0.017	0.040	0.015	3.1	3.4	0.112
*P- value*	0.04	0.48	0.57	0.09	0.03	<0.01	<0.01	<0.01

a-c Means within a column without common superscript are significantly different at the level *P* < 0.05.

1 **NDV**, Newcastle disease virus vaccine; **AIV**, avian influenza virus; **DNCB**, 2,4-dinitro 1-chlorobenzene; **PHA-P**, phytohemagglutinin-P; **H:L**, heterophil to lymphocyte ratio.

2 Experimental groups**: Control,** unchallenged; **A**, aflatoxin B_1_ challenge; **AM**, aflatoxin B_1_ challenge + multi-component toxin binder (MTB); **AMO**, aflatoxin B_1_ challenge + MTB + organic acid blend (OAB); **AC**, aflatoxin B_1_ challenge + *Clostridium perfringens* challenge; **ACM**, aflatoxin B_1_ challenge + *Clostridium perfringens* challenge + MTB; **ACMO**, aflatoxin B_1_ challenge + *Clostridium perfringens* challenge + MTB + OAB; **SEM**, standard error of the mean.

### Oxidant/Antioxidant status of serum and liver

Table 6 summarizes oxidant/antioxidant outcomes in serum and liver. Hepatic GPx activity was not altered by treatment (P > 0.05). In birds challenged with AFB₁, MTB + OAB increased serum TAC (TAC) (P < 0.05), and MTB reduced serum MDA (P < 0.05). AFB₁ decreased hepatic protein content (P < 0.05), which was improved by MTB. In the liver, AFB₁ lowered TAC and increased SOD activity and MDA concentration (P < 0.05); MTB reduced hepatic SOD and MDA, and when combined with OAB further increased TAC and lowered MDA (P < 0.05). The AFB₁ + *C. perfringens* co-challenge elevated serum and hepatic MDA and hepatic SOD activity, while decreasing hepatic protein content and TAC (P < 0.05). Both MTB and MTB + OAB mitigated these effects, with the combination generally providing the greater improvement.

**Table 6:** Effects of dietary treatments on serum and liver tissue oxidant/antioxidant status^1^ of broiler chickens challenged with aflatoxin B_1_ and *Clostridium perfringens*.

Item^2^	Serum		Liver tissue				
	TAC	MDA	Protein	TAC	SOD	GPX	MDA
	mmol/l	nmol/l	mg/g tissue	mmol/l	u/mg protein	u/mg protein	nmol/mg protein
Control	1.42^ab^	2.18^cd^	164^a^	2.58^a^	3.67^d^	2.35	1.85^c^
A	1.21^b^	2.38^c^	155^b^	1.20^c^	5.27^b^	2.66	2.26^b^
AM	1.28^b^	1.58^d^	159^ab^	1.49^bc^	4.77^c^	2.56	1.97^c^
AMO	1.65^a^	2.23^cd^	155^b^	1.68^b^	5.27^b^	2.39	1.53^d^
AC	1.26^b^	4.48^a^	145^c^	1.37^bc^	6.33^a^	2.61	2.69^a^
ACM	1.29^b^	3.55^b^	154^b^	1.57^bc^	5.29^b^	2.38	1.86^c^
ACMO	1.25^b^	2.73^c^	155^b^	1.44^bc^	5.01^bc^	2.51	2.04^bc^
SEM	0.07	0.18	2	0.09	0.11	0.13	0.07
*P- value*	0.01	<0.01	<0.01	<0.01	<0.01	0.50	<0.01

a-d Means within a column without common superscript are significantly different at the level *P* < 0.05.

1 Abbreviations: **TAC**, serum total antioxidant capacity; **MDA**, malondialdehyde; **SOD**, superoxide dismutase; **GPX**, glutathione peroxidase;

2 Experimental groups**: Control,** unchallenged; **A**, aflatoxin B_1_ challenge; **AM**, aflatoxin B_1_ challenge + multi-component toxin binder (MTB); **AMO**, aflatoxin B_1_ challenge + MTB + organic acid blend (OAB); **AC**, aflatoxin B_1_ challenge + *Clostridium perfringens* challenge; **ACM**, aflatoxin B_1_ challenge + *Clostridium perfringens* challenge + MTB; **ACMO**, aflatoxin B_1_ challenge + *Clostridium perfringens* challenge + MTB + OAB; **SEM**, standard error of the mean.

### Liver tissue morphology

**Fig. 1:**
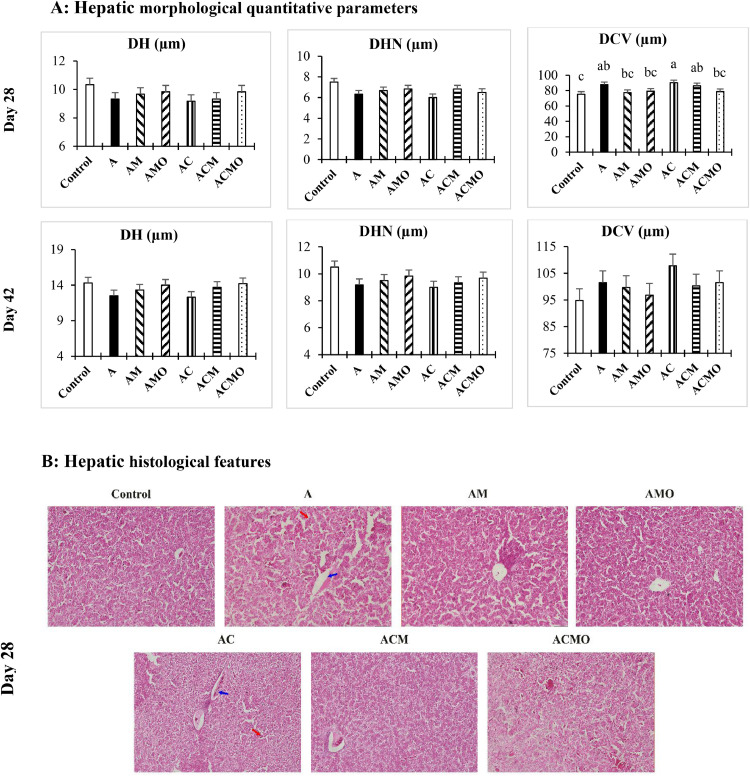
The morphological quantitative parameters (on days 28 and 42) and histological features (on day 28) of the liver in broiler chickens following co-challenged with aflatoxin B_1_ and Clostridium perfringens. For histological observation, images at a lower magnification (100 ×) are provided. In the A and AC treatment images, the red arrow marks hepatocellular necrosis and the blue arrow marks central vein distension, whereas the other groups, especially the control, AMO, and ACMO treatments, exhibit normal hepatic architecture. Abbreviation: **DH**, diameter of hepatocyte; **DHN**, diameter of hepatocyte nucleus; **DCV**, diameter of central vein. Experimental groups**: Control,** unchallenged; **A**, aflatoxin B_1_ challenge; **AM**, aflatoxin B_1_ challenge + multi-component toxin binder (MTB); **AMO**, aflatoxin B_1_ challenge + MTB + organic acid blend (OAB); **AC**, aflatoxin B_1_ challenge + *Clostridium perfringens* challenge; **ACM**, aflatoxin B_1_ challenge + *Clostridium perfringens* challenge + MTB; **ACMO**, aflatoxin B_1_ challenge + *Clostridium perfringens* challenge + MTB + OAB; **SEM**, standard error of the mean.

Fig. 1 summarizes hepatic histomorphometry at 28 and 42 days and presents representative micrographs for each treatment. Hepatocyte diameter and hepatocyte nuclear diameter at both ages, as well as CVD at day 42, did not differ among treatments (P > 0.05). In contrast, AFB₁, alone or combined with *C. perfringens*, increased CVD at day 28 (P < 0.05). Supplementation with MTB reduced CVD in AFB₁-challenged birds, and under the co-challenge a reduction was evident only when MTB was paired with OAB (Fig. 1**a**).

Histological analysis (Fig. 1**b**) corroborated the quantitative morphometric data shown in Fig. 1**a**. At days 28, livers from control birds exhibited normal lobular architecture with well-defined hepatic cords. In contrast, birds exposed to AFB₁, either alone or in combination with C*. perfringens*, displayed noticeable hepatocellular alterations, which were accompanied by a significant increase in the diameter of the central vein (P < 0.05). These vascular changes paralleled the observed increase in the DCV under AFB₁ and co-challenge conditions. Supplementation with the MTB mitigated these effects, restoring DCV values to levels comparable to those of the control group. The most pronounced recovery was seen when MTB was combined with the OAB.

### Expression of immune and antioxidant genes in jejunal tissue

**Fig. 2:**
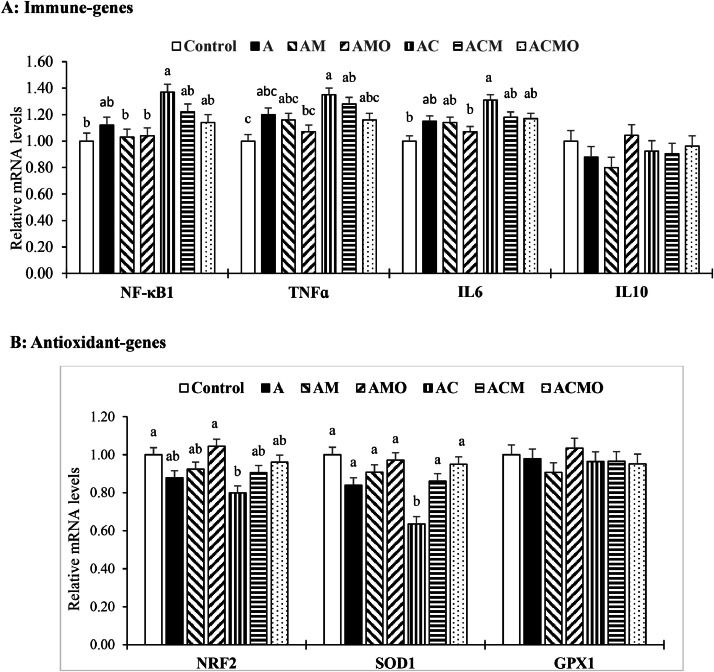
Bar charts of jejunal mRNA expression levels of immune proteins [a; nuclear factor kappa B subunit 1 (**NF-κB1**), tumor necrosis factor alpha (**TNFɑ**), interleukin 6 (**IL6**), interleukin 10 (**IL10**)] and antioxidant genes [b; nuclear factor erythroid 2-related factor 2 (**Nrf2** or **NFE2L2**), glutathione peroxidase 1 (**GPx1**), superoxide dismutase 1 (**SOD1**)] in broilers at 28 days of age. ^a–c^ Different letters in the same histogram indicate significant differences among groups according to Tukey's multiple range test (*P* < 0.05). Each bar represents the mean values and standard errors representing 6 replicates (cages) per treatment and 2 birds per treatment (n = 12 per treatment). Experimental groups**: Control,** unchallenged; **A**, aflatoxin B_1_ challenge; **AM**, aflatoxin B_1_ challenge + multi-component toxin binder (MTB); **AMO**, aflatoxin B_1_ challenge + MTB + organic acid blend (OAB); **AC**, aflatoxin B_1_ challenge + *Clostridium perfringens* challenge; **ACM**, aflatoxin B_1_ challenge + *Clostridium perfringens* challenge + MTB; **ACMO**, aflatoxin B_1_ challenge + *Clostridium perfringens* challenge + MTB + OAB; **SEM**, standard error of the mean.

Fig. 2 summarizes jejunal mRNA expression (day 28). IL-10 and GPX1 were unaffected by treatment (P > 0.05). In birds challenged with AFB₁ alone, NF-κB1, TNF-α, and IL6 tended to increase and NRF2 and SOD1 tended to decrease, but these changes were not significant (P > 0.05). The AFB₁ + *C. perfringens* co-challenge significantly upregulated NF-κB1, TNF-α, and IL6 and downregulated NRF2 and SOD1 (P < 0.05). MTB partially normalized these responses relative to the co-challenged, unsupplemented group; adding OAB to MTB did not confer further improvement for these parameters.

## Discussion

During the starter period, birds were challenged only with AFB₁, which reduced ADG. This outcome accords with established mechanisms whereby mycotoxins impair gastrointestinal function, manifesting as reduced protein synthesis, malabsorption, and diminished pancreatic enzyme secretion (e.g., amylase, trypsin), and thereby limit nutrient availability and growth (Malekinezhad et al., 2021). AFB₁ further compromises intestinal health by provoking mucosal inflammation, disrupting immune responses, elevating free-radical generation, and depressing antioxidant defenses, all of which can suppress growth (Gao et al., 2020; Mohammadi et al., 2025). In the same period, birds fed organic acids (AMO, ACMO) showed transiently poorer performance, likely due to reduced feed intake from initial palatability constraints (Table 3); this effect diminished as birds acclimated to the diets.

In subsequent phases, a concurrent *C. perfringens* challenge compounded the effects of AFB₁, depressing both ADG and FE. *C. perfringens* disrupts epithelial integrity (Ding et al., 2023), impairs digestion and absorption, and thereby compromises performance (Gharib-Naseri et al., 2019; Goo et al., 2024). In combination with AFB₁, it exacerbates necrotic enteritis and magnifies growth penalties (Abd El-Hamid et al., 2017; Cravens et al., 2013). Although the magnitude of impairment in the present study was somewhat less than reported elsewhere, the pattern was consistent: the MTB alone improved ADG under co-challenge, whereas pairing MTB with the OAB was required to recover feed efficiency.

These findings align with prior work showing that silicate and nanosilicate binders mitigate AFB₁-related growth losses (Ghazalah et al., 2021; Safaeikatouli et al., 2012), and with evidence that probiotic components can outperform clay-based binders in limiting AFB₁-induced intestinal injury (Liu et al., 2018). Organic acids likely contributed via complementary mechanisms: improving gut integrity and nutrient absorption through luminal acidification (Chowdhury et al., 2009; Saleh et al., 2025), suppressing *C. perfringens* and related pathogens (Williams, 2005), and chemically attenuating AFB₁. In acidic media, AFB₁ is converted to a β-keto acid and subsequently hydrolyzed to aflatoxin D₁, a metabolite with approximately 20-fold lower toxicity (Méndez-Albores et al., 2008; Salgado-Tránsito et al., 2011). Collectively, the adsorptive and biotransformative actions of MTB and the antimicrobial or chemical detoxification provided by OAB offer a coherent explanation for the superior performance observed with their combined use under dual challenge.

In this study, AFB₁ alone, and more markedly the combined AFB₁ + *C. perfringens* challenge, suppressed immunity. Consistent with our findings, AFB₁ has repeatedly been shown to depress cellular and humoral responses (e.g., reduced skin and toe-web reactivity to DNCB and PHA) (Arak et al., 2020), while *C. perfringens* increases intestinal inflammation and undermines immune competence in broilers (Zhang et al., 2024). In the co-challenged birds, the attenuation of the 48-h PHA toe-web response and the reduction in NDV antibody titer coincided with a shift in leukogram profiles, lower lymphocyte proportion and elevated heterophil proportion and H/L ratio, indicating stress-related immunosuppression. Such exacerbation is biologically plausible: aflatoxins increase susceptibility to bacterial, parasitic, and viral diseases (Karimi Torshizi and Sedaghat, 2023) through multiple mechanisms, including inhibition of RNA polymerase and protein synthesis, enhanced lysosomal degradation of immunoglobulins, lymphoid depletion (particularly in the bursa), and dysregulated cytokine production (Corrier, 1991). Prior reports similarly note decreased lymphocytes with increased heterophils and H/L ratio in AFB₁-exposed broilers (Rashidi et al., 2020). These mechanisms explain the compounded suppression of both cellular (PHA response) and humoral (NDV titer) immunity observed under the dual challenge.

Mitigation with MTB is consistent with literature showing that toxin binders can restore NDV titers and offset AFB₁ immunotoxicity (Lai et al., 2022) and can lessen consequences of *C. perfringens* exposure (Cravens et al., 2015). The likely basis is the complementary action of organic and inorganic sorbents plus probiotic, toxin-degrading microorganisms (e.g., *Bacillus* spp., *Bifidobacterium*), which together broaden detoxification coverage and help stabilize the gut–immune axis. The OAB may additionally contribute via chemical attenuation of AFB₁, lactic and acetic acids from *Lactobacillus casei paracasei* can convert AFB₁ to the far less toxic B₂a (Simões et al., 2023), and through anti-inflammatory effects (reduced IL-2/IFN-γ; (Cai et al., 2025)). In our data, OAB improved immune indices under AFB₁ alone, whereas benefits were limited under *C. perfringens* co-challenge, suggesting pathogen burden and tissue injury may have exceeded the scope of acidifier support. Even so, the combined MTB + OAB approach provided broader protection than MTB alone across several immune responses under the dual challenge.

### Oxidant/antioxidant balance

In the present study, hepatic protein content declined with AFB₁ exposure and fell further under the combined AFB₁ + *C. perfringens* challenge. This agrees with reports that dietary AFB₁ lowers liver protein in broilers (Chen et al., 2023). The liver is a primary target of AFB₁; hepatotoxic effects include bile duct epithelial hyperplasia, hepatocellular degeneration and necrosis, and parenchymal nodular changes with inflammatory cell infiltration (Patil et al., 2014; Yohannis et al., 2025). Such lesions, together with sinusoidal dilation and edema, can reduce functional tissue mass per gram of liver, plausibly explaining the lower measured protein concentration despite similar organ volume.

AFB₁ also imposes oxidative stress by generating reactive oxygen species and depleting antioxidant reserves. It promotes lipid peroxidation (Chen et al., 2022) and elevates hepatic MDA (Chen et al., 2023), and may impair normal antioxidant function by disrupting digestion/absorption of lipids and vitamins C, E, and A (Shirzadi et al., 2024). Consistent with these mechanisms, we observed increased MDA and reduced TAC in serum and liver; these disturbances were accentuated by *C. perfringens*, which can add to the oxidative/inflammatory burden. Although some studies have found decreased GPx and SOD activities with AFB₁ (Chen et al., 2022; Gowda et al., 2008; Yarru et al., 2009), we detected higher hepatic SOD activity (with a non-significant rise in GPx) under AFB₁ alone and the dual challenge. Similar compensatory elevations have been reported: AFB₁ increased SOD in porcine jejunal mucosa (Choi et al., 2025) and elevated hepatic SOD and glutathione in broilers (Chen et al., 2024). Elevated SOD can reflect a defensive response to oxidative stress (Sulzberger et al., 2017) and is often interpreted as a biomarker of increased oxidative load (Anwar et al., 2025). The observed increase in hepatic SOD activity and the concomitant decrease in jejunal SOD1 mRNA expression following AFB_1_ and Clostridium perfringens co-challenge can be attributed to isoform-specific regulation of SOD enzymes. While SOD1 is the primary cytosolic form, SOD2 (mitochondrial) and SOD3 (extracellular) isoforms may be upregulated under oxidative stress to better manage reactive oxygen species (Sulzberger et al., 2017; Zheng et al., 2023). The increase in hepatic SOD activity likely reflects a compensatory antioxidant response in the liver, whereas the reduction in SOD1 expression in the jejunum may indicate a shift toward the upregulation of other SOD isoforms, such as SOD2, in response to the oxidative load. These findings highlight the complex regulation of antioxidant systems in response to dual oxidative challenges, where the liver and jejunum may utilize different isoforms to mitigate oxidative damage (Anwar et al., 2025).

Nutritional countermeasures aligned with these pathophysiological patterns. Organic acids have been shown to raise antioxidant enzyme activities (SOD, GPx) (Lin et al., 2023), and, together with toxin binders, to limit aflatoxicosis (Assar et al., 2018). In fish, toxin binders increased TAC and multiple antioxidant enzymes (Phudkliang et al., 2025). Accordingly, in our trial the MTB improved oxidative indices, likely by adsorbing AFB₁ and limiting systemic exposure, while its combination with OAB further enhanced TAC and lowered MDA, plausibly via chemical attenuation of AFB₁ and reduced *C. perfringens* colonization. Overall, these results support a dual strategy that both restricts toxin bioavailability and moderates the oxidative and inflammatory milieu.

In our study, AFB₁ exposure widened the hepatic central vein at day 28, with greater dilation under the combined AFB₁ + *C. perfringens* challenge; by contrast, hepatocyte and nuclear diameters showed a downward trend in both AFB₁-challenged and AFB₁ + *C. perfringens* challenged groups but did not differ significantly from control. Similar central venous congestion has been reported in other species: AFB₁ enlarged the central vein in common carp (Al-Rubaiy et al., 2018) and, in Wistar rats, induced central venous congestion and dilation, vacuolar degeneration, necrotic foci, bile duct hyperplasia, and portal congestion (Ali et al., 2021). Mechanistically, hepatic bioactivation of AFB₁ to the 8,9-epoxide generates DNA/protein adducts and oxidative injury, plausibly contributing to sinusoidal stasis and venous dilation (Taranu et al., 2020; Cheng et al., 2023c).

Mitigation with the MTB is consistent with prior work showing reduced hepatic lipid accumulation, necrosis, and nuclear hypertrophy in AFB₁-challenged broilers given binders (Zabiulla et al., 2021). Under the dual challenge, MTB combined with OAB was superior to MTB alone in normalizing central vein diameter, likely reflecting the added antimicrobial and acidifying actions of OAB (limiting *C. perfringens* overgrowth) and its potential to attenuate AFB₁ chemically. Together, these effects reduce hepatic toxic load and vascular congestion, improving hepatocellular architecture.

The gut-associated lymphoid tissue comprises multiple immune cell types that release pro- and anti-inflammatory mediators to maintain intestinal homeostasis and provide a barrier against pathogen invasion (Broom and Kogut, 2018). Accordingly, under challenge, chickens modulate cytokine profiles to counter pathogenic insults. In our study, AFB_1_ alone numerically increased expression of NFκB and the pro-inflammatory cytokines TNF-α and IL-6, and decreased expression of the antioxidant genes NRF2 and SOD1; under the combined AFB_1_ and *C. perfringens* challenge, these changes became significant, indicating a stronger inflammatory response and suppression of antioxidant defenses. This pattern agrees with reports that AFB_1_ upregulates NF-κB, TNF-α, and IL-6 while reducing SOD (Mohammadi et al., 2024), and that aflatoxin G1 increases TNF-α in THP-1 macrophages (Zheng et al., 2023). NF-κB is a key transcription factor in inflammatory pathways, driving induction of TNF-α and IL-6 (Liu et al., 2017). IL-6 can trigger immune responses, mitigate toxin-induced damage, and promote release of other inflammatory mediators (Hirano, 2021). Elevated IL-6 mRNA has been observed with aflatoxin exposure (Long et al., 2016) and during necrotic enteritis caused by *C. perfringens* (Park et al., 2008). Consistently, *C. perfringens* activates the NF-κB pathway and increases jejunal inflammatory gene expression in broilers (Tang et al., 2022; Zhang et al., 2024).

Both *in vitro* and *in vivo* studies show that AFB_1_ induces ROS and oxidative stress (Kövesi et al., 2020). NRF2 is a central transcription factor maintaining redox balance in response to oxidative challenge, increasing transcription of antioxidant enzymes to neutralize free radicals (Wang et al., 2018). Mycotoxins can downregulate NRF2 via multiple signaling routes (NRF2/HO-1, HIF-1α, PI3K/Akt, AhR), thereby reducing NRF2 and downstream detoxification genes (Kozieł et al., 2021; Ye et al., 2025). Similarly, AFB_1_ decreases NRF2 expression in the livers of broilers (Chen et al., 2024) and rabbits (Zhang et al., 2023). The reduced expression of antioxidant genes together with elevated serum and hepatic malondialdehyde (Table 6) in birds co-challenged with AFB_1_ and *C. perfringens* indicates a disrupted oxidant–antioxidant balance and induction of oxidative stress.

Physical–biological binder combinations have been reported to reduce inflammation and improve antioxidant status in broilers by adsorbing and degrading toxins (Guo et al., 2023). Thus, the improved regulation of immune and antioxidant gene expression in the jejunum with MTB likely reflects effective toxin adsorption and the contribution of probiotic bacteria in the formulation; by binding and biotransforming AFB_1_ and limiting *C. perfringens* colonization, MTB reduced inflammatory signaling and normalized gene expression. Although organic acids can attenuate intestinal inflammation by improving microbial composition and epithelial function (Ding et al., 2023), they did not further enhance gene-expression regulation in our conditions, possibly because MTB alone achieved the maximal improvement attainable for these parameters.

## Conclusions

In conclusion, the combined challenge of AFB_1_ and *C. perfringens* caused more significant impairments in growth performance, immune response, and oxidant/antioxidant status of broiler chickens compared to the AFB_1_ challenge alone, leading to increased oxidative stress and inflammation. While the use of a MTB was effective in mitigating many of the detrimental effects of AFB_1_, the addition of an OAB supplement did not consistently provide additional benefits. However, OAB was necessary to improve key economic traits and liver health under co-challenge conditions. Based on these findings, it is recommended to use OAB in combination with MTB for broiler chickens facing simultaneous challenges of AFB_1_ and *C. perfringens*.

## CRediT authorship contribution statement

**Maryam Karimi Zandi:** Writing – original draft, Visualization, Investigation, Data curation, Conceptualization. **Hassan Shirzadi:** Writing – review & editing, Writing – original draft, Supervision, Software, Investigation, Formal analysis. **Hossein Ali Ghasemi:** Writing – review & editing, Writing – original draft, Validation, Supervision, Investigation, Conceptualization. **Mohammad Amir Karimi Torshizi:** Writing – review & editing, Validation, Software, Investigation. **Kamran Taherpour:** Writing – review & editing, Resources, Project administration, Conceptualization. **Enayat Rahmatnejad:** Writing – review & editing, Validation, Software, Methodology, Conceptualization.

## Disclosures

The authors state that they have no conflicts of interest related to the study.

## Appendix Supplementary materials

Image, image 1 dummy alt text
