# Supplementary figures and images for: Protective effects of a multicomponent toxin binder and organic acid blend on feed efficiency, oxidative status, hepatic histology, and jejunal immune–antioxidant responses in broilers co-challenged with aflatoxin B1 and Clostridium perfringens
Source: Poult Sci. 2026 Jan 29;105(4):106546. doi: 10.1016/j.psj.2026.106546 (PMC12919270; doi:10.1016/j.psj.2026.106546)

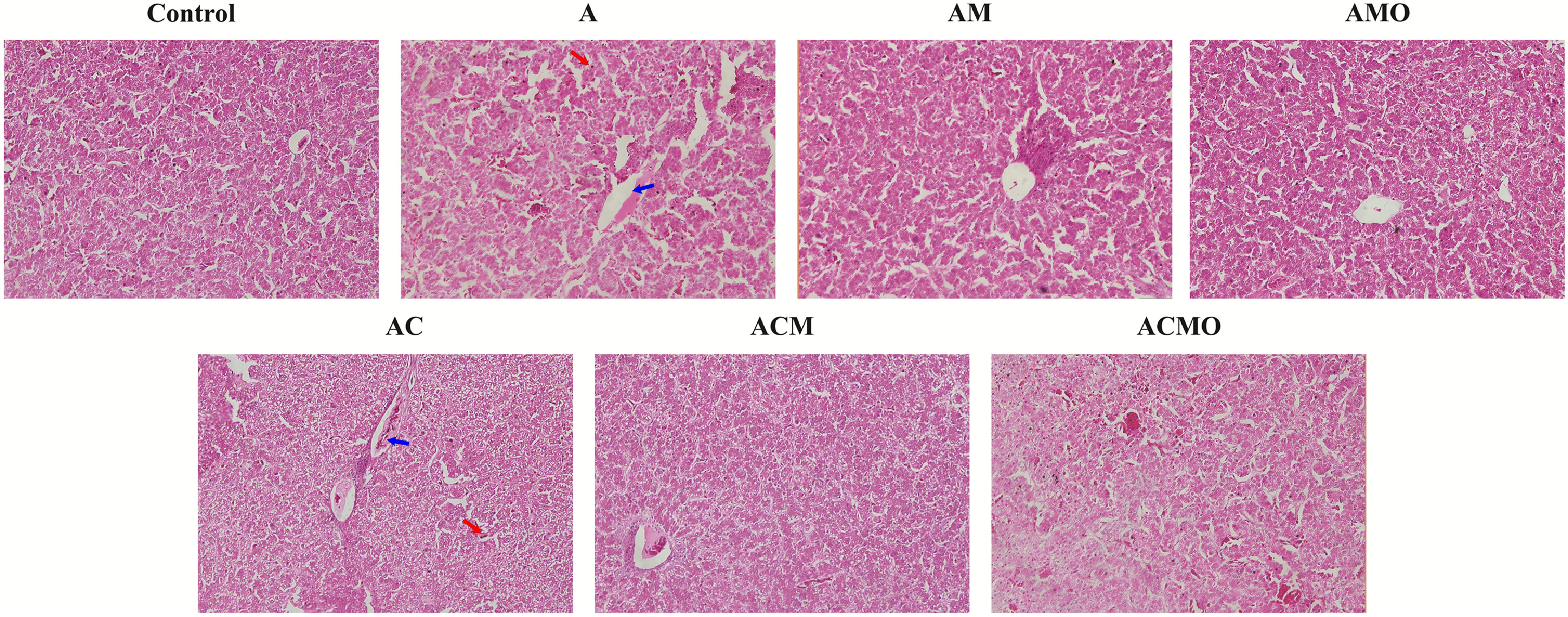

Supplement: Supplementary file 1 [file mmc1.jpg]
